# Supplementary material for: It’s not you, it’s me: A thematic analysis of written causal attributions for favorable and unfavorable feedback on different types of Instagram image sharing
Source: PLoS One. 2026 May 11;21(5):e0346413. doi: 10.1371/journal.pone.0346413 (PMC13160335; doi:10.1371/journal.pone.0346413)
Supplement: Attachment — Submitted filename: Response to Reviewers.docx [file pone.0346413.s002.docx]

Dear Dr. Shiva Ram Male,

I have addressed both your comments and those of the reviewer regarding my manuscript titled *“It's not you, it's me: A thematic analysis of written causal attributions for favorable and unfavorable feedback on different types of Instagram image sharing”* (PONE-D-25-26407). I appreciate the thoughtful feedback, as it has helped to strengthen the manuscript. My detailed responses to the comments are provided below.

Regards,

Malinda Desjarlais, Ph.D.

**Section 1 - Editor Comments**

Journal Requirements:

When submitting your revision, we need you to address these additional requirements.

1. Please ensure that your manuscript meets PLOS ONE's style requirements, including those for file naming. The PLOS ONE style templates can be found at

<https://journals.plos.org/plosone/s/file?id=wjVg/PLOSOne_formatting_sample_main_body.pdf> and

[https://journals.plos.org/plosone/s/file?id=ba62/PLOSOne_formatting_sample_title_authors_affiliations.pdf](https://track.editorialmanager.com/CL0/https:%2F%2Fjournals.plos.org%2Fplosone%2Fs%2Ffile%3Fid=ba62%2FPLOSOne_formatting_sample_title_authors_affiliations.pdf/1/010f019a0c5c8f25-596035b1-d32c-4784-b712-e8172dcd9f15-000000/rLi4w9NYRqJjzJZDzQRlOR0F9Tmxbj7ytkBC6rfZXAg=234).

**Response**: The manuscript and file naming has been checked to ensure it meets PLOS ONES’s style requirements.

2. You indicated that you had ethical approval for your study. In your Methods section, please ensure you have also stated whether you obtained consent from parents or guardians of the minors included in the study or whether the research ethics committee or IRB specifically waived the need for their consent.

**Response**: the following (in red) has been added to the manuscript (pages 10-11) to clarify the consent process:

Participants arrived at the lab. Participants completed an informed consent process and subsequently provided written consent. In accordance with the institution’s ethical guidelines, university students, irrespective of age, may independently provide informed consent for research participation. Accordingly, parental consent was not required for participants under 18 years of age.

3. We note that there is identifying data in the Supporting Information file <file name>. Due to the inclusion of these potentially identifying data, we have removed this file from your file inventory. Prior to sharing human research participant data, authors should consult with an ethics committee to ensure data are shared in accordance with participant consent and all applicable local laws.

Data sharing should never compromise participant privacy. It is therefore not appropriate to publicly share personally identifiable data on human research participants. The following are examples of data that should not be shared:

-Name, initials, physical address

-Ages more specific than whole numbers

-Internet protocol (IP) address

-Specific dates (birth dates, death dates, examination dates, etc.)

-Contact information such as phone number or email address

-Location data

-ID numbers that seem specific (long numbers, include initials, titled “Hospital ID”) rather than random (small numbers in numerical order)

Data that are not directly identifying may also be inappropriate to share, as in combination they can become identifying. For example, data collected from a small group of participants, vulnerable populations, or private groups should not be shared if they involve indirect identifiers (such as sex, ethnicity, location, etc.) that may risk the identification of study participants.

Additional guidance on preparing raw data for publication can be found in our Data Policy (<https://journals.plos.org/plosone/s/data-availability#loc-human-research-participant-data-and-other-sensitive-data>) and in the following article: <http://www.bmj.com/content/340/bmj.c181.long>.

Please remove or anonymize all personal information (<specific identifying information in file to be removed>), ensure that the data shared are in accordance with participant consent, and re-upload a fully anonymized data set. Please note that spreadsheet columns with personal information must be removed and not hidden as all hidden columns will appear in the published file.

**Response**: After reviewing the content of the supplementary information, I felt that it did not contribute meaningfully to the manuscript. These documents simply summarized the characteristics of the participants and their quantitative responses to the questions in a different format that what was presented in the manuscript. Therefore, I decided to remove all the supplementary information documents. If researchers require access to the data to verify the findings the data would be made available upon request (as outlined below).

4. We note that you have indicated that there are restrictions to data sharing for this study. For studies involving human research participant data or other sensitive data, we encourage authors to share de-identified or anonymized data. However, when data cannot be publicly shared for ethical reasons, we allow authors to make their data sets available upon request. For information on unacceptable data access restrictions, please see <http://journals.plos.org/plosone/s/data-availability#loc-unacceptable-data-access-restrictions>.

Before we proceed with your manuscript, please address the following prompts:

a) If there are ethical or legal restrictions on sharing a de-identified data set, please explain them in detail (e.g., data contain potentially identifying or sensitive patient information, data are owned by a third-party organization, etc.) and who has imposed them (e.g., a Research Ethics Committee or Institutional Review Board, etc.). Please also provide contact information for a data access committee, ethics committee, or other institutional body to which data requests may be sent.

b) If there are no restrictions, please upload the minimal anonymized data set necessary to replicate your study findings to a stable, public repository and provide us with the relevant URLs, DOIs, or accession numbers. Please see <http://www.bmj.com/content/340/bmj.c181.long> for guidelines on how to de-identify and prepare clinical data for publication. For a list of recommended repositories, please see <https://journals.plos.org/plosone/s/recommended-repositories>. You also have the option of uploading the data as Supporting Information files, but we would recommend depositing data directly to a data repository if possible.

Please update your Data Availability statement in the submission form accordingly.

**Response**: A complete data file containing all participant responses cannot be shared publicly for ethical reasons. The consent form that participants signed stated that *“Your individual responses will not be published in any form.”* To comply with the standards approved by the Mount Royal University Human Research Ethics Board, the data file can only be provided upon request by emailing the author directly. These requests must go through the author because Mount Royal University does not have an office responsible for managing such data access.

5. Please include captions for your Supporting Information files at the end of your manuscript, and update any in-text citations to match accordingly. Please see our Supporting Information guidelines for more information: <http://journals.plos.org/plosone/s/supporting-information>.

**Response**: As Supporting Information has been removed, captions are no longer needed.

6. If the reviewer comments include a recommendation to cite specific previously published works, please review and evaluate these publications to determine whether they are relevant and should be cited. There is no requirement to cite these works unless the editor has indicated otherwise.

**Response**: not applicable

7. Please review your reference list to ensure that it is complete and correct. If you have cited papers that have been retracted, please include the rationale for doing so in the manuscript text, or remove these references and replace them with relevant current references. Any changes to the reference list should be mentioned in the rebuttal letter that accompanies your revised manuscript. If you need to cite a retracted article, indicate the article’s retracted status in the References list and also include a citation and full reference for the retraction notice.

**Response**: The references were reviewed and ensure correct.

Additional Editor Comments (if provided):

Dear Author,

Kindly go through the revisions suggested by the Reviewer

**Section 2 - Reviewers' comments:**

Reviewer's Responses to Questions

**Comments to the Author**

5. Review Comments to the Author

Reviewer #1: your manuscript titled “It’s not you, it’s me: A thematic analysis of written causal attributions for favorable and unfavorable feedback on different types of Instagram image sharing.” This is a timely and thoughtfully designed study that offers valuable insights into how individuals interpret social feedback on self-presentational content across social media platforms. Below are my comments and suggestions aimed at helping you refine and strengthen your manuscript.

Major Strengths:

Novel Contribution: The manuscript extends attribution theory into the domain of social media feedback, which is relatively underexplored. The focus on real-time personal reactions rather than imagined scenarios is a significant advancement.

Methodological Rigor: The experimental use of fabricated feedback and the inclusion of three image types (selfie, elsie, meme) is innovative and well-executed.

Thematic Clarity: The reflexive thematic analysis is robust, with well-categorized themes and illustrative participant responses.

Integration with Theory: The use of attribution theory, sociometer theory, self-objectification, and self-verification theories helps provide depth to your findings.

**Response**: Thank you for your support.

Suggestions for Improvement:

1. Theoretical Clarity: While self-serving bias is mentioned, the observed deviation (internal attributions even for unfavorable feedback) could benefit from deeper theoretical reflection. Could self-objectification override typical self-protective mechanisms? A more direct engagement with the contradiction between attribution theory and observed patterns would strengthen your discussion.

**Response**: Thank you for pointing this out—I agree that the discussion would benefit from a deeper theoretical reflection. In response, I have expanded the discussion to more thoroughly address the theoretical implications of the findings. The following additions were incorporated into the discussion to strengthen this section:

First, the discussion of the pattern “attributing favorable feedback to controllable factors and unfavorable to uncontrollable” was expanded. The following was added to the discussion (pages 20-21):

Such attributional tendencies align closely with patterns associated with shame. When negative appearance feedback is received, it is more likely to evoke shame, an emotion rooted in the perception that something is wrong with the whole self rather than with a specific behavior [43]. Shame reliably promotes an attributional pattern characterized by global and uncontrollable causes – perceiving negative outcomes as stemming from broad, stable, and unchangeable personal flaws (e.g., “I’m unattractive,” “There is something wrong with me”) rather than specific, situational factors (e.g., poor lighting or camera angle). These global and uncontrollable attributions have been documented in body and appearance contexts specifically [e.g., 44].

To build on this point, the discussion of participants’ non-use of societal attitudes toward selfies was moved earlier in the discussion section. This shift allowed for a more coherent and theoretically grounded explanation of how self-objectification may have overshadowed typical self-protective mechanisms in this context. Accordingly, the following material was added to the manuscript immediately after the non-use of societal attitudes section of the discussion (pages 21-22):

Objectification theory suggests that the internalized perspective manifests as habitual and vigilant self-monitoring of their appearance for flaws [37, 38]. Thus, even when aware of negative societal attitudes toward selfie-posting, individuals may default to appearance-focused internal attributions when their image is evaluated. Additionally, research on the imagined audience shows that social media users think primarily in terms of specific viewers rather than society at large, which is perceived as too distant to be causally relevant [45]. Participants may have viewed the immediate audience, not cultural stereotypes, as the primary evaluators, reducing the likelihood that societal narratives were used as self-protective explanations.

Overall, these findings highlight how selfies and elsies shared on SNSs not only makes appearance salient but also fosters internalized appearance evaluation through the nature of the feedback received. When the self is viewed as an object under gaze, situational defences might be less salient or psychological accessible, and traditional self-serving logic may not hold, increasing the likelihood of internalized negative attributions.

2. Sample and Generalizability: sample (majority young females) is clearly reported. However, consider expanding the discussion on how this demographic bias may have influenced the attribution patterns, particularly in relation to appearance-based evaluations and societal beauty norms.

**Response**: Although the sample composition (76% women) was briefly acknowledged in the original discussion, I agree that this demographic imbalance could have influenced participants’ causal attributions, particularly given the relevance of self-objectification theory to appearance-based evaluations. In response, I have integrated this point more explicitly earlier in the discussion. The paragraph (page 20) has been revised accordingly, with the new additions highlighted in red:

Women in cultures that place a strong emphasis on appearance are particularly likely to become objectified – treated as objects to be looked at and evaluated based on how they look [37, 38]. Over time, they internalize this external perspective and start viewing and judging their physical appearance from a third-person perspective. Research shows that contexts prompting individuals to consider how others will evaluate their appearance reliably heighten self-objectification during SNS use [39-41]. Experimental research reveals that the act of taking selfies, regardless of whether they are shared, can increase state self-objectification and body dissatisfaction compared to taking photos of objects [26], among men and women [41]. Self-objectification has also been shown to increase among women after receiving favorable appearance-based comments on their selfies [42]. Given that (women) participants believed their selfie or elsie would be shared with an audience comprised of their peers and evaluated for feedback, the study context likely activated this objectifying gaze. As a result, internal causal attributions for social feedback were largely centered on their physical appearance.

Additionally, the sample composition is now acknowledged as a limitation of the study. The following was added to the Limitations and future directions section (page 24):

As previously noted, the results must be considered in the context of the sample characteristics, particularly the overrepresentation of whom, who comprised 76% of the participants. Prior research suggests that women, particularly in Western cultural contexts, experience higher levels of self-objectification, engage more frequently in appearance-based self-evaluation, and may respond differently to both positive and negative appearance feedback than men. As a result, the attributional patterns observed in this study may reflect gendered experiences of social media use and appearance-related evaluation. Future research should examine whether similar patterns emerge in more gender-balanced samples or in samples that allow for meaningful comparisons across gender and/or cultural identities.

3. Interpretation of Meme Feedback: The findings around meme feedback (predominantly external attributions) are intriguing. You may consider further elaboration using the online disinhibition effect or content detachment frameworks to explain this distancing.

**Response**: Although the online disinhibition effect was mentioned in the original discussion, I agree that it required deeper theoretical consideration in the context of the causal attributions for the meme condition. In response, the section dedicated to the findings for the meme condition has been revised to more fully explain the underlying mechanisms of the online disinhibition effect and how these mechanisms may account for the observed attribution patterns. The revised text appears on page 23, with the added content highlighted in red:

Across both favorable and unfavorable outcomes, participants overwhelmingly attributed responses to meme posts to external factors. This pattern for meme-related feedback can be understood through the lens of online disinhibition. According to the online disinhibition effect, anonymity and invisibility fosters psychological distance and a sense that one’s online actions are detached from the real self [49]. In this state, users experience dissociation between one’s online behaviour and one’s offline identity, perceived as what goes on online is less reflective of the real self, resulting in less emotional investment and more detached from outcomes. For the meme condition, participants had minimal involvement in creating the content, contributing only by selecting a pre-existing meme, and the posts were shared without identifying information. The meme became a non-self-referential object, which according to the literature would generate low self-objectification [26, 41], resulting in lower personal accountability for reactions. As a result, participants likely experienced the meme as an object independent of the self, making audience reactions feel directed at the humor of the meme rather than at them personally. This sense of detachment was likely amplified by anonymity, which can reduce the identity stake in outcomes [49]. Consequently, participants may have perceived the imagined audience, not themselves, as the central agent in evaluating the post, prompting participants to externalize the source of the feedback they received.

4. Perceived Societal Attitudes vs. Attribution: A strong point of your study is that participants acknowledged negative societal perceptions toward selfies but did not attribute unfavorable feedback to those broader norms. This disconnect is important and could be more explicitly explored in the discussion section.

**Response**: This section of the discussion has been moved earlier in the manuscript so that it is now more clearly integrated with the explanation of the causal attribution patterns for selfies (now positioned on page 21), rather than appearing as an add-on at the end of that section. As noted above, this portion of the discussion has also been expanded to clarify how self-objectification may overshadow typical self-protective attributional biases in this context.

Minor Recommendations:

1. Standardize terminology: consider consistently using either “self-images” or “selfies and elsies” for clarity.

**Response**: Consistent terminology is now used throughout the manuscript. I refer to the mentioned images as selfies and elsies. The revised terminology is highlighted in red within the manuscript.

2. The methods section includes some repetition (e.g., feedback condition explanations); tightening this would improve flow.

**Response**: The redundant content pertaining to the method has been removed from the Current Study section for succinctness. This section focuses on a brief introduction to the study design and emphasizes the research questions. It now reads as follows (pages 7-8):

Employing an experimental design in which participants were led to believe that their selfie, elsie, or selected meme had received either favorable or unfavorable feedback, the present study seeks to advance our understanding of social feedback processes by examining: *What attributional explanations do individuals formulate for the social responses their posts elicit, particularly with respect to the locus-of-control dimension?* Furthermore, to contextualize potential attributional patterns and extend existing research on personal evaluations of others’ self-presentation, the study also investigates a secondary question: *What are individuals’ perspectives of society’s opinions toward those who post selfies versus elsies?*

The above replaced the previous version, which read:

The present study aims to advance our understanding of social feedback by examining: *What types of attributions do individuals make for the social responses their posts receive?* Participants were randomly assigned to one of three conditions (i.e., to take a selfie, to have an elsie taken by the researcher, or to select a meme from provided options -- which served as a non-appearance-focused comparison group), under the belief that their image would be posted to a university-affiliated Instagram account for 5 minutes. They were made to believe that their peers at the university would be responding to their post to provide a relevant imaginary audience context. After the 5-minute posting period, participants were told they had received favorable or unfavorable responses and then asked to provide causal attributions for the type of feedback they received using open-ended and Likert-style questions.

Participants were also asked to report their perception of societal attitudes toward selfies compared to elsies, to explore whether these perceptions may have influenced their attributions. Given the focus on whether participants attributed feedback of the imagined audience to internal or external factors, the emphasis was placed on their perception of broader societal attitudes rather than their own personal opinions. Due to the prevailing negative view of selfie-posters compared to elsie-posters in the extant literature, participants in the selfie condition had an opportunity to attribute unfavorable feedback to broader societal dislike of this form of self-presentation. To provide a potential context for attribution patterns and to extend existing research on personal evaluations of others’ self-presentation, the present study also explored: *What are individuals’ perspectives of society’s opinions of those who post selfies versus elsies?*

Overall, the present study documented and compared the types of causal attributions individuals made between favorable and unfavorable feedback across self-images (selfies and elsies) and memes on Instagram, with particular attention to the dimension of locus of control. Additionally, commonalities and individual differences in perceived societal attitudes toward different types of self-images were documented.

3. Please proofread for minor typographical issues (e.g., "appearancerelated" should be "appearance-related").

**Response**: The manuscript has been proofread. Thank you for drawing my attention to this.

4. A procedural diagram illustrating the study design could enhance reader comprehension.

**Response**: Fig. 1 was created to summarize the procedure (see below). Reference to this figure was added in on page 11 (“See Fig1 for summary of the procedure.”)

5. If possible, consider sharing your thematic codebook in the Supporting Information for transparency and reproducibility.

**Response**: Ultimately, the codebook is contained within Tables 1, 2, and 3 of the manuscript. To make this clearer, I have updated the table headings to align with the terminology used in the description of the qualitative analysis. Accordingly, “Details” has been changed to “Subthemes” and “Themes” has been changed to “Overarching Themes”.

Final Remarks i felt:
Overall, this is a well-written and engaging manuscript that makes a meaningful contribution to the literature on digital self-presentation and social cognition. I believe it will be of great interest to both psychological and media studies audiences. I recommend very minor revision to address the points above, which will further enhance the clarity and impact of your work.
Best regards,
Reviewer
